# Anesthetic-Induced Disruption of Amino Acid and Carnitine Profiles: A Metabolomic Comparison of Propofol and Thiopental in Hepatocytes

**DOI:** 10.3390/ph18081221

**Published:** 2025-08-19

**Authors:** Veli F. Pehlivan, Basak Pehlivan, Erdogan Duran, Ismail Koyuncu, Hamza Erdogdu

**Affiliations:** 1 Department of Anesthesiology and Reanimation, Faculty of Medicine, Harran University, Şanlıurfa 63200, Turkey; bpehlivan@harran.edu.tr (B.P.); dreduran@harran.edu.tr (E.D.); 2 Department of Medical Biochemistry, Faculty of Medicine, Harran University, Şanlıurfa 63200, Turkey; ismailkoyuncu1@gmail.com; 3 Department of Biostatistics, Faculty of Medicine, Harran University, Şanlıurfa 63200, Turkey; hamzaerdogdu@harran.edu.tr

**Keywords:** Propofol, Thiopental, metabolomics, amino acids, carnitine, hepatocytes, AML12 cells

## Abstract

**Background/Objectives:** Propofol and Thiopental are widely used anesthetic agents, yet their cumulative and high-dose effects on hepatic metabolism remain insufficiently characterized. This study aimed to evaluate the impact of supra-therapeutic concentrations of these agents on carnitine and amino acid metabolism in AML12 hepatocytes, with a focus on their toxicometabolic profiles. **Methods:** AML12 mouse hepatocytes were exposed to escalating concentrations (2.5–500 µg/mL) of Propofol and Thiopental to assess cytotoxicity. IC_50_ values (~255 µg/mL for both) were determined, and two high-dose concentrations (100 µg/mL and 200 µg/mL) were selected for metabolic profiling. Cell viability was assessed via the MTT assay. Intracellular carnitine and amino acid levels were quantified using LC-MS/MS. Statistical analyses included one-way ANOVA with post hoc tests, unpaired *t*-tests, and effect size estimations (Cohen’s d). **Results:** Propofol significantly suppressed carnitine metabolism in a dose-dependent manner, with a 79% reduction in free carnitine (C0), indicative of impaired mitochondrial β-oxidation. Thiopental, however, preserved or partially restored several acylcarnitines, including C16:1. While both agents reduced intracellular amino acid levels, 200 µg/mL Thiopental partially restored key metabolites such as glutamine, alanine, and histidine. Propofol exhibited broader metabolic suppression. Effect size analysis further confirmed the stronger inhibitory impact of Propofol. **Conclusion:** Although the concentrations used exceed typical clinical plasma levels, they may reflect prolonged or high-dose exposure scenarios observed in ICU settings. The findings highlight distinct toxicometabolic signatures for each agent and underscore the utility of metabolite profiling in modeling anesthetic-induced hepatic stress and guiding anesthetic selection in vulnerable populations.

## 1. Introduction

Intravenous anesthetic agents are widely employed in clinical anesthesia due to their rapid onset, ease of titration, and favorable pharmacokinetic profiles, making them suitable for both surgical procedures and intensive care sedation protocols [1,2]. Although generally considered safe, prolonged exposure or administration at high doses has been associated with cumulative metabolic and toxic effects, particularly in metabolically active organs such as the liver [3,4,5]. Propofol, due to its lipophilic nature, exhibits rapid onset of action, whereas Thiopental, a barbiturate derivative, possesses a longer half-life and a higher potential for accumulation. However, the toxic or modulatory effects of these agents on hepatocellular metabolic pathways, especially at the mitochondrial level, remain incompletely understood [6]. Given the liver’s central role in maintaining metabolic homeostasis, elucidating how these anesthetic agents influence amino acid and carnitine metabolism, which are critical for mitochondrial energy production and redox balance, holds significant clinical importance.

The liver plays a pivotal role in the biotransformation of anesthetic agents and the maintenance of systemic metabolic equilibrium, largely through the regulation of cellular stress responses [7,8,9]. Hepatocytes contribute to both energy generation and oxidative balance through pathways such as amino acid metabolism and carnitine-mediated β-oxidation [7,8,9,10]. Disruptions to these pathways particularly under conditions of high-dose or prolonged anesthetic exposure may lead to mitochondrial dysfunction, increased oxidative stress, and impaired cellular recovery.

Characterizing anesthetic-induced alterations in amino acid and carnitine profiles is therefore critical to understanding the hepatic metabolic impact of these agents. Such insights can inform efforts to preserve perioperative metabolic stability, assess the risk of hepatotoxicity, and develop safer anesthetic strategies for clinical practice [10,11,12,13,14].

In recent years, the potential of anesthetic agents to reprogram cellular metabolism particularly under stress conditions or in metabolically vulnerable patients has emerged as a compelling area of investigation [15]. Alterations in amino acid and acylcarnitine profiles are increasingly recognized as sensitive biomarkers of mitochondrial integrity and cellular metabolic stress [16,17]. However, comparative studies evaluating the distinct metabolic effects of different anesthetic agents remain limited.

In our previous work, we demonstrated that cumulative exposure to Propofol, Thiopental, and Dexmedetomidine induces significant cytotoxicity, oxidative stress, and apoptosis in AML12 hepatocytes [17]. Building upon these findings, the present study specifically evaluates the effects of equivalent and increasing concentrations of Propofol and Thiopental on intracellular amino acid and carnitine profiles. This approach enables a detailed assessment of the metabolic safety landscape associated with each anesthetic agent. By analyzing the differential effects of both drugs on mitochondrial fatty acid metabolism, energy homeostasis, and amino acid balance, the study aims to reveal distinct cellular metabolic response patterns.

We hypothesize that high-dose exposure to Propofol and Thiopental will induce significant alterations in intracellular amino acid and carnitine metabolite levels in AML12 hepatocytes. These changes are expected to reflect either suppressive or compensatory effects on mitochondrial function. Specifically, Propofol is anticipated to elicit a more widespread and dominant metabolic suppression profile, whereas Thiopental may exhibit a more heterogeneous pattern by selectively preserving or increasing certain metabolites. These distinctions are expected to elucidate drug-specific mitochondrial response mechanisms under anesthetic-induced metabolic stress.

The primary aim of this study is to quantitatively compare the effects of high cumulative concentrations of Propofol and Thiopental on amino acid and carnitine levels in AML12 hepatocytes. In this context, the study seeks to characterize the cellular metabolic responses elicited by each intravenous anesthetic agent and to identify potential risks of metabolic imbalance, particularly in clinically critical scenarios such as prolonged infusion or high-dose administration. The findings are intended to contribute to metabolomics-based frameworks for guiding anesthetic selection and to support the development of strategies aimed at preserving perioperative metabolic stability.

## 2. Results

### 2.1. AML12 Cell Viability

**Figure 1:**
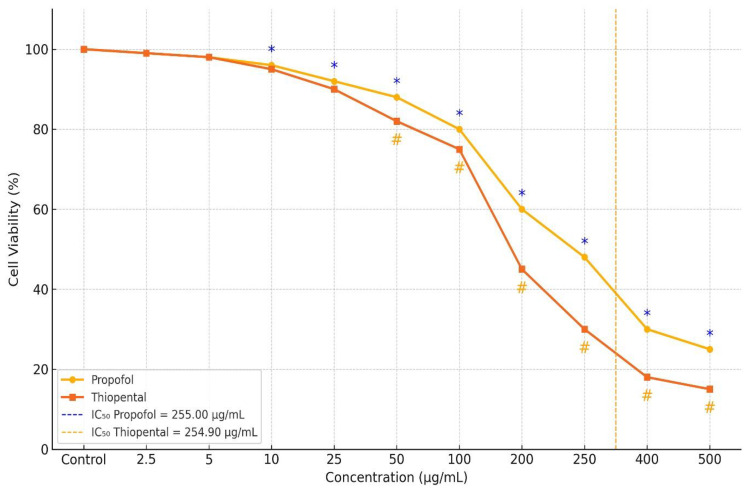
Dose-dependent changes in viability of AML12 hepatocytes following Propofol and Thiopental exposure [17]. Figure depicts the percentage of viable cells after treatment with increasing concentrations of each anesthetic agent. Both Propofol and Thiopental significantly decreased cell viability at concentrations ≥ 100 µg/mL compared to the control group (*p* < 0.001). Notably, at concentrations of 200, 250, and 500 µg/mL, Propofol induced significantly less cytotoxicity than Thiopental (*p* < 0.001). IC_50_ values were calculated as 255.008 µg/mL for Propofol and 254.904 µg/mL for Thiopental. Data are presented as mean ± standard deviation (SD). * indicates *p* < 0.001 compared to the control group for Propofol; # indicates *p* < 0.001 compared to the control group for Thiopental.

As reported in our previous study (Figure 1) [17], the cytotoxic effects of Propofol and Thiopental on AML12 hepatocytes were evaluated using the MTT assay, and those results were also referenced in the current study. The analysis revealed a dose-dependent reduction in cell viability for both agents.

The calculated IC_50_ values were 255.008 µg/mL for Propofol and 254.904 µg/mL for Thiopental. At concentrations of 100 µg/mL and 400 µg/mL, both agents exhibited significantly greater cytotoxicity compared to the control group (*p* < 0.001), with no notable difference between the two drugs. However, at concentrations of 200, 250, and 500 µg/mL, Propofol exhibited significantly lower cytotoxicity than Thiopental. The reductions in cell viability observed at these concentrations were statistically significant both compared to control and between the two agents (*p* < 0.001).

### 2.2. Carnitine Profile Following Propofol and Thiopental Exposure in AML12 Hepatocytes

**Figure 2:**
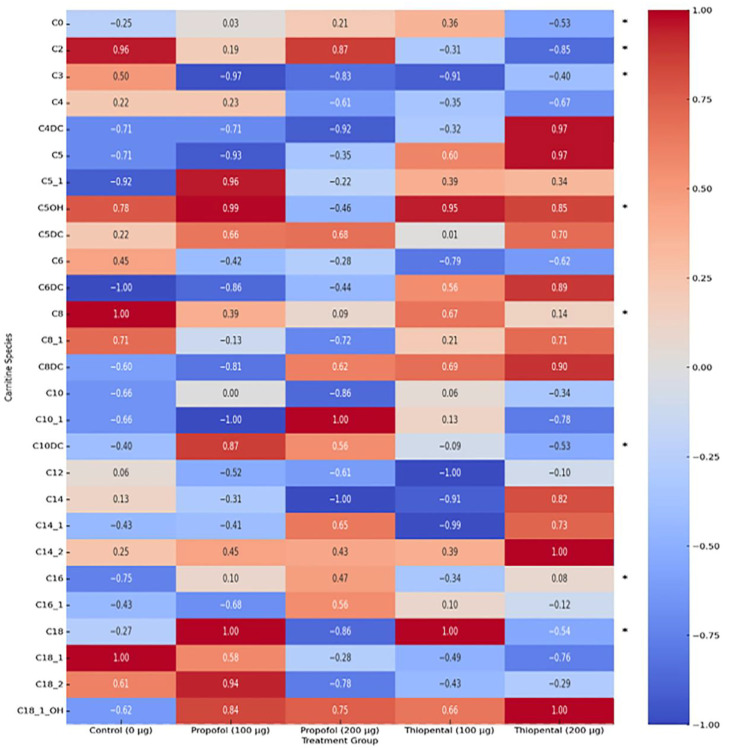
Heatmap of intracellular carnitine and acylcarnitine profiles in AML12 hepatocytes after Propofol and Thiopental exposure. Heatmap showing the normalized intracellular levels of carnitine and acylcarnitine species in AML12 hepatocytes following exposure to cumulative doses (100 µg and 200 µg) of Propofol and Thiopental. Data were normalized to a range of –1 to +1. (*) indicate metabolites that exhibited statistically significant changes compared to the control group (*p* < 0.05).

Table 1 presents the intracellular concentrations of selected carnitine and acylcarnitine species in AML12 hepatocytes following high cumulative exposure to Propofol and Thiopental (100 µg/mL and 200 µg/mL) compared to untreated controls (0 µg/mL). These representative metabolites were selected based on their biological relevance and the magnitude of change observed. The results reveal pronounced anesthetic-induced metabolic stress responses, along with dose-dependent disruptions in mitochondrial fatty acid metabolism and carnitine homeostasis. A complete dataset including all quantified carnitine and acylcarnitine metabolites is provided in Supplementary Table S1. These alterations are also visually summarized in Figure 2, which displays a heatmap of intracellular carnitine and acylcarnitine profiles, highlighting the differential metabolic impact of each anesthetic agent.

**Free Carnitine (C0) and Acetylcarnitine (C2):** Both anesthetics significantly reduced intracellular C0 and C2 levels, with the most prominent effects observed in the 200 µg/mL Propofol group (*p* < 0.001). Propofol induced an approximate 79% decrease in C0, indicating substantial impairment of the carnitine shuttle system essential for mitochondrial β-oxidation.

**Long-Chain Acylcarnitines (C14, C16, and C18):** Propofol led to significant reductions in long-chain acylcarnitines such as C14, C16, and C18 (*p* < 0.001), suggesting impaired transport of long-chain fatty acids into the mitochondria. In contrast, Thiopental produced less pronounced reductions and even showed partial restoration or increases in some species (e.g., C16:1) at the 200 µg/mL dose, highlighting its more selective effect on mitochondrial fatty acid metabolism relative to Propofol.

**Medium-Chain Acylcarnitines (C4, C5, C6, C8, C10, and C12):** Both anesthetics caused a general reduction in medium-chain acylcarnitines, with Propofol exerting more pronounced and dose-dependent effects. For example, 200 µg/mL Propofol led to marked decreases in C8 and C10, whereas Thiopental’s effects on these metabolites were more moderate. This suggests that Propofol exerts broader mitochondrial suppression, while Thiopental affects more specific pathways.

**Other Acylcarnitines (C3, C4-OH, C5-OH, C10:1, C12:1, C14:1, C14:2, C16:1, C18:1, and C18:2):** The profiles of these metabolites exhibited complex patterns for both anesthetics. Propofol generally reduced most of these species, while Thiopental showed increases in some (e.g., C16:1). These findings suggest that different anesthetics target distinct metabolic pathways and that these metabolites may serve as specific biomarkers of anesthetic-induced metabolic disturbances.

### 2.3. Amino Acid Profile Following Propofol and Thiopental Exposure in AML12 Hepatocytes

**Figure 3:**
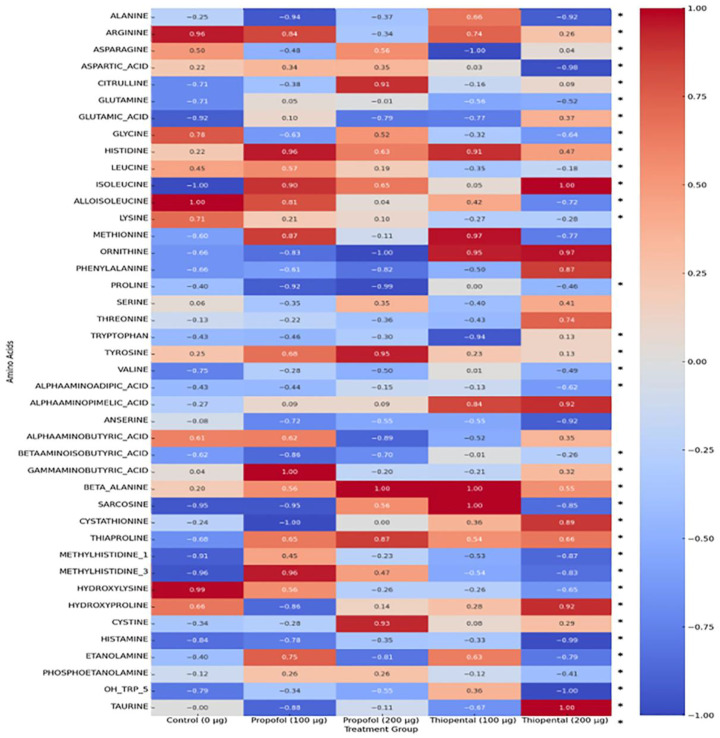
Normalized heatmap of amino acid profiles in AML12 hepatocytes exposed to Propofol and Thiopental. Heatmap representation of normalized amino acid concentrations in AML12 hepatocytes after exposure to 100 µg and 200 µg doses of Propofol and Thiopental. Values are scaled between 1 and +1. (*) indicate statistically significant changes compared to the control group (*p* < 0.05).

Table 2 presents the intracellular concentrations of selected free amino acid metabolites in AML12 hepatocytes following exposure to high cumulative doses of Propofol and Thiopental (100 µg/mL and 200 µg/mL) compared to untreated control cells (0 µg/mL). These representative metabolites were chosen based on their biological relevance and the magnitude of observed alterations. The findings highlight agent-specific changes in amino acid metabolism, which may have implications for hepatic protein synthesis, redox balance, and energy production. A comprehensive dataset including all quantified amino acids is provided in Supplementary Table S2. Additionally, Figure 3 offers a visual summary of the overall amino acid profiles, displaying a normalized heatmap that illustrates distinct patterns of metabolic disruption induced by each anesthetic agent.

**Overall Amino Acid Reduction:** Both anesthetics caused a general decrease in intracellular amino acid concentrations, possibly reflecting enhanced protein catabolism or impaired amino acid synthesis. However, the effect of Propofol was more pronounced and dose-dependent. For instance, 200 µg/mL Propofol caused significant reductions in several amino acids, including alanine, glutamine, and glycine, whereas Thiopental at the same dose showed milder effects.

**Glutamine and Alanine:** These amino acids play key roles in hepatic metabolism. Propofol induced substantial reductions in both, while 200 µg/mL Thiopental resulted in partial restoration or increases, suggesting that Thiopental may better preserve hepatic metabolic responses under stress conditions compared to Propofol.

**Branched-Chain Amino Acids (BCAAs) (Valine, Leucine, and Isoleucine):** These essential amino acids are crucial for energy production and protein synthesis. Both anesthetics reduced BCAA levels, with Propofol again showing a more prominent and dose-dependent suppression. This suggests that Propofol may exert broader inhibitory effects on BCAA metabolism.

**Other Amino Acids:** The profiles of other amino acids (e.g., histidine, tryptophan, and tyrosine) showed complex responses. While Propofol generally decreased the levels of these metabolites, Thiopental caused increases in some (e.g., histidine). These variations reinforce the notion that anesthetics exert agent-specific effects on distinct metabolic pathways, and that these metabolites may serve as biomarkers of targeted disruptions.

### 2.4. Integrated Metabolic Interpretation

**Figure 4:**
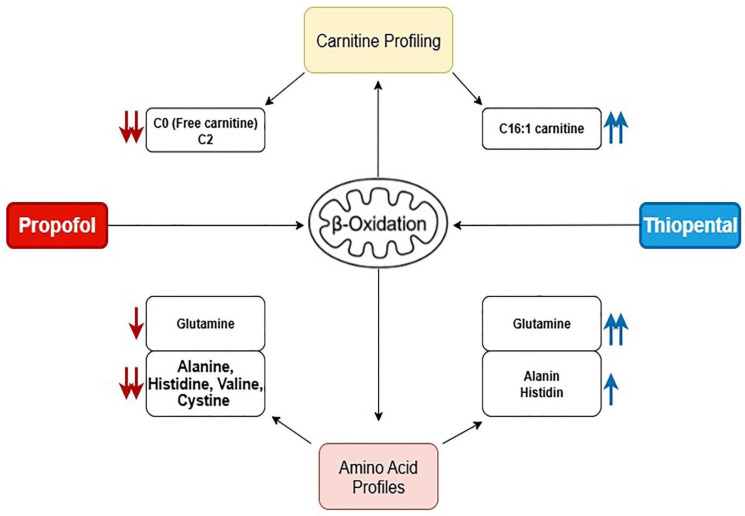
Schematic representation of the proposed mechanisms by which Propofol and Thiopental alter mitochondrial β-oxidation and amino acid metabolism in hepatocytes. Propofol predominantly suppresses free carnitine and amino acid levels, impairing fatty acid transport and mitochondrial energy production, whereas Thiopental shows selective restoration of specific acylcarnitines and amino acids, suggesting a more adaptive metabolic response. (Red arrows (↓) indicate significant decreases, while blue arrows (↑) represent increases in specific carnitine or amino acid levels. The arrow colors also reflect the treatment groups: red arrows denote changes associated with Propofol exposure, and blue arrows indicate changes associated with Thiopental exposure).

To visually summarize the differential metabolic effects of Propofol and Thiopental, we present an integrated schematic (Figure 4) that depicts their impact on mitochondrial β-oxidation and amino acid metabolism. As shown, Propofol induces widespread suppression of free carnitine, long-chain acylcarnitines, and key amino acids such as glutamine and alanine, reflecting impaired fatty acid transport and mitochondrial energy production. In contrast, Thiopental demonstrates partial preservation or even restoration of certain acylcarnitines and amino acids, suggesting a more adaptive metabolic response. These pathway-specific alterations are consistent with the quantitative data presented in Table 1 and Table 2 and Figure 2 and Figure 3 and may offer mechanistic insight into anesthetic-specific metabolic stress responses.

### 2.5. Comparative Effect Size Analysis of Amino Acid and Carnitine Alterations

**Figure 5:**
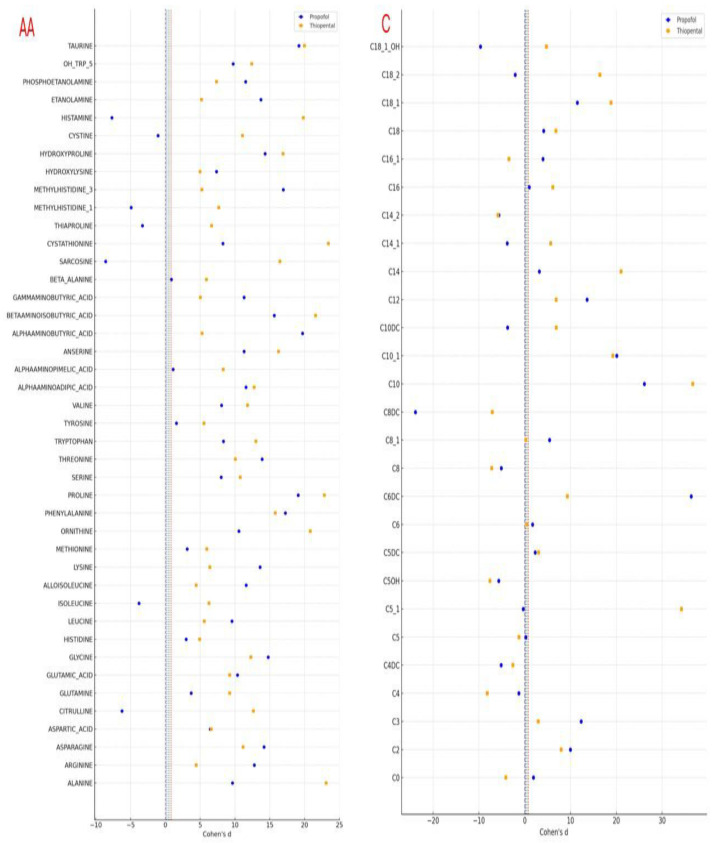
Effect size analysis (Cohen’s d) of amino acid and carnitine profile changes in AML12 cells after Propofol and Thiopental exposure. This combined forest plot illustrates the effect sizes (Cohen’s d) for the changes in intracellular amino acid (left) and carnitine (right) levels in AML12 hepatocytes following exposure to cumulative doses of Propofol and Thiopental. Blue circles indicate Propofol, and orange squares represent Thiopental. Vertical reference lines indicate conventional thresholds for small (d = 0.2), medium (d = 0.5), and large (d = 0.8) effect sizes. Negative values denote a reduction compared to the control group (AA: amino acid, C: carnitine).

Following the observed statistically significant changes in amino acid and carnitine levels after Propofol and Thiopental exposure in AML12 hepatocytes, it was deemed essential to evaluate the magnitude of these differences not solely in terms of *p*-values but also in terms of biological and clinical relevance. Therefore, Cohen’s d values were calculated to quantify the effect sizes associated with each metabolite change and to enable direct comparisons between the two anesthetic agents. This approach allowed for a more nuanced interpretation of the metabolic impact beyond significance testing, highlighting distinct differences in metabolic response patterns between Propofol and Thiopental. The calculated Cohen’s d values are illustrated in a combined forest plot (Figure 5), offering a comprehensive visualization of both the magnitude and direction of changes in amino acid and carnitine profiles.

These findings emphasize that, beyond merely achieving statistical significance, Propofol and Thiopental exert quantitatively distinct impacts on hepatocellular amino acid and carnitine metabolism. The implications of these metabolic disruptions and the potential underlying mechanisms are discussed in the following section.

## 3. Discussion

This study comprehensively investigated the differential effects of Propofol and Thiopental on amino acid and carnitine metabolism in AML12 hepatocytes. Our findings demonstrate that these two widely used intravenous anesthetic agents modulate hepatic metabolic pathways through distinct mechanisms, with important implications for clinical practice.

Propofol exhibited a broader and more suppressive effect on carnitine metabolites, significantly impairing mitochondrial β-oxidation. The marked reductions in free carnitine (C0) and acetylcarnitine (C2) levels suggest that Propofol may inhibit the transport and oxidation of fatty acids within mitochondria. This observation aligns with clinical manifestations of Propofol Infusion Syndrome (PRIS), which is characterized by metabolic complications such as lactic acidosis and rhabdomyolysis, especially under prolonged or high-dose Propofol exposure [4,7]. PRIS is a rare but serious condition associated with mitochondrial dysfunction. Our results support previous research suggesting that Propofol can directly inhibit the mitochondrial respiratory chain and disrupt fatty acid oxidation [2,6].

In contrast, Thiopental exerted more selective effects on carnitine metabolites, with partial restoration or even increases in specific acylcarnitines (e.g., C16:1). This suggests that Thiopental may have a less globally suppressive effect on mitochondrial function or may differentially modulate specific metabolic pathways. The amino acid profiles also revealed distinct patterns; while Propofol induced widespread reductions, Thiopental preserved or even restored key amino acids such as glutamine and alanine, which play central roles in gluconeogenesis, redox balance, and the urea cycle. Thiopental’s ability to preserve these metabolites may reflect a more favorable metabolic profile in terms of protein synthesis and energy production.

These differences may be attributed to the distinct pharmacodynamic properties of the two agents. Propofol is known to inhibit mitochondrial respiratory chain complexes I and II, leading to reduced ATP synthesis and increased oxidative stress [2,6]. Thiopental, on the other hand, primarily acts through GABAergic mechanisms, and its mitochondrial effects may be less pronounced or mechanistically distinct [18]. Our findings suggest that the metabolic effects of anesthetics are not only dose- and time-dependent but are also closely related to their specific mechanisms of action.

The use of AML12 cells as an in vitro model is a major strength of this study. AML12 is a well-characterized murine hepatocyte line that closely mimics key metabolic functions of the liver [19,20]. This model provides valuable insights into the fundamental metabolic effects of anesthetics at the cellular level. However, the translational applicability of in vitro findings remains limited. The complex physiology and regulatory mechanisms of the human liver cannot be fully recapitulated in vitro. A limitation of this study is the use of AML12 cells, which, while widely accepted, represent a simplified hepatocytic model lacking the multicellular complexity and endocrine regulation of the human liver. Moreover, while our findings suggest anesthetic-induced mitochondrial dysfunction based on metabolomic patterns, further validation using direct assays such as Seahorse-based OCR/ECAR measurements or CPT1/2 expression profiling is warranted. Future studies should validate these findings in clinical settings and assess the long-term effects of anesthetic exposure on perioperative metabolic stability using human serum/plasma or hepatic amino acid profiling.

The concentrations used in this study (100 µg/mL and 200 µg/mL) were based on IC_50_ values determined in our previous cytotoxicity experiments [17]. While these doses exceed therapeutic plasma concentrations (typically 3–6 µg/mL for Propofol [21]), they were selected to model the cumulative effects of high-dose exposure, as may occur in prolonged infusions or critical care scenarios. Although the concentrations used in this study exceed typical clinical plasma levels, they may be representative of prolonged or high-dose exposure scenarios encountered in ICU settings. Thus, the findings primarily reflect the toxicometabolic profile associated with supra-therapeutic anesthetic concentrations. These findings underscore the potential risk of metabolic disruption during high-dose or long-term anesthetic administration. This is particularly relevant for critically ill patients requiring extended sedation or those with pre-existing metabolic disorders such as hepatic insufficiency, obesity, diabetes, or mitochondrial disease.

Although this study focused on two commonly used anesthetic agents, future studies comparing additional agents such as etomidate, ketamine, or volatile anesthetics would enhance the scope of metabolic safety profiling and broaden clinical applicability. Inclusion of multiple drug comparisons may help delineate agent-specific metabolic signatures and clarify the role of underlying molecular pathways in anesthetic-induced metabolic stress.

Additionally, while this study demonstrated pronounced reductions in several amino acids, the potential protective role of amino acid supplementation (e.g., glutamine or branched-chain amino acids) was not investigated. This concept merits future exploration, particularly in light of our findings that Thiopental partially preserved certain key metabolites. Whether supplementation could mitigate anesthetic-induced metabolic suppression and accelerate recovery in clinical settings remains a compelling avenue for further research.

Given the consistent reductions in mitochondrial β-oxidation intermediates and amino acids, a schematic model of the proposed disruptions in hepatic metabolism is provided (Figure 4). This visual representation summarizes the likely pathways affected by Propofol and Thiopental, including impairments in fatty acid transport, mitochondrial oxidation, and amino acid recycling. Such schematics may facilitate mechanistic understanding and foster hypothesis generation for future experimental validation.

Understanding how anesthetic agents influence perioperative metabolic stability and hepatotoxic risk is essential to enhancing patient safety and improving postoperative outcomes. This consideration is especially critical for metabolically vulnerable individuals, such as those with liver dysfunction, metabolic syndrome, or inherited metabolic disorders. Recent evidence suggests that amino acid and carnitine profiling may provide valuable biomarkers for detecting anesthetic-induced metabolic stress [22,23,24]. These assessments could be integrated into perioperative protocols to identify patient susceptibility and tailor anesthetic management accordingly. Given the agent specific metabolic disruptions observed, perioperative amino acid and carnitine profiling may serve as useful biomarkers to predict anesthetic tolerance in metabolically vulnerable patients. Prospective clinical studies are warranted to explore the predictive value of such profiling in guiding personalized anesthetic management.

## 4. Materials and Methods

### 4.1. Cell Line and Culture Conditions

AML12 mouse hepatocyte cells were obtained from the American Type Culture Collection (ATCC, Manassas, VA, USA) [19,20]. This cell line was selected as an in vitro model due to its capacity to mimic hepatic metabolic functions and provide reproducible results in metabolic assays. Cells were cultured in Dulbecco’s Modified Eagle Medium: Nutrient Mixture F-12 (DMEM/F12) and supplemented with 10% fetal bovine serum (FBS), 1% penicillin–streptomycin (P/S), and 1% L-glutamine. Cultures were maintained at 37 °C in a humidified atmosphere containing 5% CO_2_.

Cells were passaged at a ratio of 1:2 or 1:3 using 0.25% trypsin and 0.03% ethylenediaminetetraacetic acid (EDTA) solution according to ATCC guidelines. Unused cells were cryopreserved in a freezing medium containing 95% complete growth medium and 5% dimethyl sulfoxide (DMSO), initially stored at –80 °C, and later transferred to liquid nitrogen for long-term preservation.

### 4.2. Drug Administration to Cells

#### Drug Exposure and Cytotoxicity Assessment (MTT Assay)

AML12 cells were seeded into sterile 96-well plates at a density of 1 × 10^4^ cells/well. After 24 h, the culture medium was refreshed. Ten concentrations (0, 2.5, 5, 10, 25, 50, 100, 200, 250, and 400–500 µg/mL) of Propofol and Thiopental were selected for cytotoxicity screening. Each concentration was tested in triplicate. Cells in the control group received no drug treatment. Following drug exposure, cells were incubated for an additional 24 h at 37 °C in a 5% CO_2_ atmosphere.

After incubation, the medium was aspirated, and cytotoxicity was assessed using the colorimetric MTT assay (3-(4,5-dimethylthiazol-2-yl)-2,5-diphenyltetrazolium bromide). Absorbance was measured at 570 nm (reference wavelength: 690 nm) using a microplate reader Thermo Multiskan GO (Thermo Fisher Scientific, Waltham, MA, USA). Dose–response curves were generated, and half-maximal inhibitory concentration (IC_50_) values were calculated. Based on the IC_50_ values determined in our previous study [17], concentrations of 100 µg/mL and 200 µg/mL were selected for subsequent experiments involving both Propofol and Thiopental. These concentrations fall within the high-dose exposure range and are suitable for modeling the cumulative metabolic effects of anesthetics. Clinically relevant plasma concentrations of Propofol typically range from 3 to 6 µg/mL [21]; the higher concentrations used in this study were chosen to simulate the potential cumulative toxicity observed in critical clinical scenarios such as prolonged infusions or high-dose administration.

### 4.3. Cell Homogenization and Carnitine Profiling

Following drug exposure, cells were detached from the culture surface using trypsinization, collected, and centrifuged at 1000 rpm for 5 min to obtain a cell pellet. Cold lysis buffer was added to the pellet, and homogenization was performed using a TissueLyser system (Qiagen, Hilden, Germany). The homogenate was centrifuged again, and the supernatant was carefully collected.

A 5 µL aliquot of the supernatant was spotted onto Guthrie cards, air-dried overnight at 25 °C, and then cut into small pieces and transferred into clean tubes. Carnitine profiling was performed based on previously published methods by La Marca et al. [25], Azzari et al. [26], and Sağlık et al. [27]. Prior to analysis, 200 µL of internal standard solution was added to each sample, which was then dried under a nitrogen stream for 30 min. Next, 60 µL of a derivatization reagent (butanolic-HCl) was added, and the mixture was incubated at 60 °C for 30 min, followed by another drying step under nitrogen. Finally, the samples were reconstituted in 100 µL of mobile phase (acetonitrile/methanol/formic acid) and analyzed using a Shimadzu LC-MS/MS 8040 system (Shimadzu Corporation, Kyoto, Japan) under positive electrospray ionization (ESI+) mode using multiple reaction monitoring (MRM). Quantitative measurements were obtained using the instrument’s software based on comparison with internal standards.

A detailed list of LC-MS/MS parameters, including MRM transitions, collision energies, and ion source settings for each analyte, is provided in Supplementary Table S3A (Carnitines) to enhance methodological transparency and reproducibility.

### 4.4. LC-MS/MS Analysis of Free Amino Acid Profiles

Analysis of free amino acids was conducted using a modified version of the protocol described by Çelik et al. [28], in conjunction with a commercial kit (Trimaris Biotechnology, Istanbul, Türkiye), and performed on a Shimadzu LCMS-8045 system. This derivatization-based method is designed for the quantitative determination of free amino acids.

Analytes were prepared in 0.1 M HCl and included 20 isotopically labeled amino acids (^13C and ^15N). A 100 µL aliquot of each sample was mixed with internal standard solution. A basic organic buffer prepared in propanol was then added to adjust the pH and facilitate efficient derivatization. This step also induced protein precipitation.

Next, a derivatizing agent consisting of 5% alkyl chloroformate in a chloroform/isooctane mixture was added. After 3 min of incubation at room temperature, derivatized amino acids were extracted into the upper organic phase via centrifugation. A 1 µL aliquot of this phase was injected into the LC-MS/MS system.

Due to the esterification process, the molecular weights of the amino acids increased, enhancing volatility and signal intensity in mass spectrometry. Chromatographic separation was performed on a Trimaris Amino Acid LC-MS/MS column (C18 reverse-phase, 250 mm × 2 mm, 3 µm particle size). Mobile phase A consisted of a water/methanol/1 M ammonium formate mixture (85:14:1), while mobile phase B was pure methanol. Detection of amino acids was achieved using positive ion electrospray ionization (ESI) and MRM acquisition. A detailed list of LC-MS/MS parameters, including MRM transitions, collision energies, and ion source settings for each analyte, is provided in Supplementary Table S3B (Amino Acids) to enhance methodological transparency and reproducibility.

### 4.5. Statistical Analysis

All statistical analyses were conducted using IBM SPSS Statistics version 24.0 (IBM Corp., Armonk, NY, USA). The normality of data distribution was assessed using both the Kolmogorov–Smirnov and Shapiro–Wilk tests. Continuous variables were presented as mean ± standard deviation (SD). One-way analysis of variance (ANOVA) was employed to compare mean amino acid and carnitine concentrations at different cumulative doses of Propofol and Thiopental (0 µg/mL, 100 µg/mL, and 200 µg/mL). When overall significance was detected, Tukey’s post hoc test was used for multiple comparisons to identify specific group differences. Direct comparisons between Propofol and Thiopental at equivalent doses (100 µg/mL and 200 µg/mL) were performed using independent sample Student’s *t*-tests. A two-tailed *p*-value less than 0.05 was considered statistically significant for all tests. Where applicable, effect sizes (Cohen’s d) were calculated to estimate the magnitude of observed differences.

## 5. Conclusions

In conclusion, this study demonstrates the distinct and agent-specific metabolic disruptions induced by Propofol and Thiopental in AML12 hepatocytes, particularly in amino acid and carnitine homeostasis. By employing high-dose concentrations reflective of cumulative or prolonged use in intensive care settings, this model provides a relevant toxicometabolic framework for assessing the cellular impact of anesthetics. The observed alterations (more pronounced with Propofol) suggest that anesthetic agents differentially influence hepatic metabolic pathways, with potential implications for mitochondrial function, protein synthesis, and redox regulation. These findings underscore the importance of incorporating metabolomics-informed strategies into anesthetic decision making. Further clinical studies are warranted to evaluate whether perioperative amino acid and carnitine profiling could serve as predictive tools for anesthetic tolerance, particularly in metabolically vulnerable or critically ill patients.

## Figures and Tables

**Table 1:** Representative alterations in key carnitine and acylcarnitine metabolites in AML12 hepatocytes following high-dose Propofol and Thiopental exposure.

	Control		Propofol					Thiopental					Propofol vs. Thiopental	
Dose	0 µg		100 µg		200 µg			100 µg		200 µg			100 µg	200 µg
	Mean	Std. Deviation	Mean	Std. Deviation	Mean	Std. Deviation	*p*-Value	Mean	Std. Deviation	Mean	Std. Deviation	*p*-Value	*p*-Value	*p*-Value
C0	0.4115	0.0151	0.1477	0.0032	0.0862	0.0026	**<0.001**	0.1452	0.0073	0.2403	0.0045	**<0.001**	0.625	**<0.001**
C2	0.8540	0.0183	0.5362	0.0072	0.5081	0.0078	**<0.001**	0.3631	0.2064	0.7415	0.0226	**0.006**	0.220	**<0.001**
C3	0.1938	0.0068	0.1404	0.0090	0.1390	0.0076	**<0.001**	0.1225	0.0820	0.1779	0.0088	0.238	0.726	**0.004**
C4	0.0426	0.0051	0.0428	0.0027	0.0319	0.0097	0.142	0.1576	0.0907	0.0284	0.0023	**0.044**	0.093	0.575
C5OH	0.0637	0.0051	0.0578	0.0054	0.0502	0.0046	**0.046**	0.0581	0.0590	0.0551	0.0051	0.954	0.992	0.286
C8	0.0060	0.0016	0.0075	0.0023	0.0022	0.0015	**0.032**	0.0376	0.0092	0.0037	0.0036	**0.001**	**0.005**	**0.541**
C10	0.0127	0.0063	0.0090	0.0050	0.0066	0.0032	0.384	0.0404	0.0175	0.0078	0.0019	0.020	**0.040**	0.618
C10DC	0.0359	0.0005	0.0289	0.0016	0.0239	0.0004	**<0.001**	0.0216	0.0211	0.0234	0.0029	0.361	0.582	0.778
C12	0.0017	0.0030	0.0031	0.0008	0.0002	0.0004	0.215	0.0202	0.0133	0.0004	0.0007	**0.038**	0.156	0.741
C16	0.0148	0.0019	0.0084	0.0013	0.0110	0.0006	**0.004**	0.0161	0.0122	0.0079	0.0029	0.391	0.384	0.203
C16_1	0.0029	0.0023	0.0040	0.0014	0.0018	0.0013	0.343	0.0195	0.0095	0.0008	0.0009	**0.013**	**0.049**	0.350
C18	0.0114	0.0006	0.0081	0.0009	0.0085	0.0009	0.004	0.0141	0.0198	0.0067	0.0021	0.736	0.648	0.241

Intracellular concentrations (mean ± SD) of representative carnitine and acylcarnitine metabolites in AML12 hepatocytes following cumulative exposure to Propofol and Thiopental (100 µg/mL and 200 µg/mL) compared to untreated controls (0 µg/mL). Statistically significant changes (*p* < 0.05) are highlighted. Propofol-treated cells were used as the reference group for comparative analyses. (Full metabolite profiles are provided in Supplementary Table S1).

**Table 2:** Representative alterations in key amino acid metabolites in AML12 hepatocytes following high-dose Propofol and Thiopental exposure.

	Control		Propofol					Thiopental					Propofol vs. Thiopental	
Dose	0 µg		100 µg		200 µg			100 µg		200 µg			100 µg	200 µg
	Mean	Std. Deviation	Mean	Std. Deviation	Mean	Std. Deviation	*p*-Value	Mean	Std. Deviation	Mean	Std. Deviation	*p*-Value	*p*-Value	*p*-Value
ALANINE	19.75	1.30	7.63	0.50	6.58	0.43	**<0.001**	7.72	0.51	8.20	0.54	**<0.001**	0.851	**0.015**
ARGININE	20.37	1.43	4.93	0.35	13.50	0.94	**<0.001**	0.77	0.05	5.90	0.41	**<0.001**	**0.002**	**<0.001**
ASPARTIC_ACID	6.77	0.54	2.42	0.19	2.18	0.17	**<0.001**	3.46	0.28	3.47	0.28	**<0.001**	**0.006**	**0.002**
GLUTAMINE	556.80	50.05	323.50	29.08	687.39	61.78	**<0.001**	428.34	38.50	711.47	63.95	**0.002**	**0.020**	0.663
GLUTAMIC_ACID	42.59	4.06	13.73	1.31	11.92	1.14	**<0.001**	13.99	1.33	13.15	1.25	**<0.001**	0.823	0.277
HISTIDINE	3.46	0.37	1.66	0.18	2.21	0.24	**0.001**	2.20	0.23	2.51	0.27	**0.005**	**0.034**	0.220
ORNITHINE	1.11	0.37	0.77	0.26	0.94	0.31	0.476	2.97	0.99	2.39	0.80	0.059	**0.020**	**0.043**
PHENYLALANINE	3.43	1.18	1.44	0.49	1.83	0.63	0.053	1.76	0.60	2.25	0.77	0.137	0.511	0.500
TRYPTOPHAN	2.12	0.29	1.04	0.14	1.28	0.18	**0.002**	1.21	0.17	1.32	0.18	**0.005**	0.263	0.783
TYROSINE	8.96	1.29	3.90	0.56	5.23	0.75	**0.001**	4.88	0.70	5.91	0.85	**0.005**	0.134	0.352
VALINE	11.18	2.14	4.57	0.87	5.43	1.04	**0.003**	5.42	1.04	5.78	1.11	**0.006**	0.339	0.704
GAMMAMINOBUTYRIC_ACID	0.53	0.13	0.00	0.00	0.35	0.09	**0.001**	0.01	0.00	0.39	0.10	**0.001**	**0.007**	0.611
METHYLHISTIDINE_1	0.00	0.00	0.03	0.01	0.03	0.01	**0.004**	0.00	0.00	0.04	0.01	**<0.001**	**0.004**	0.407
METHYLHISTIDINE_3	0.01	0.00	0.02	0.00	0.00	0.00	**0.014**	0.00	0.00	0.00	0.00	**0.001**	**0.005**	**0.016**
CYSTINE	2.61	0.39	0.20	0.03	1.56	0.23	**<0.001**	0.40	0.06	1.03	0.15	**<0.001**	**0.007**	**0.030**
HISTAMINE	0.00	0.00	0.01	0.00	0.00	0.00	**0.001**	0.00	0.00	0.01	0.00	**0.004**	**0.024**	**0.002**
ETANOLAMINE	0.75	0.15	0.19	0.04	0.51	0.10	**0.002**	0.35	0.07	0.22	0.05	**0.002**	**0.029**	**0.012**

Intracellular concentrations (mean ± SD) of representative amino acid metabolites in AML12 hepatocytes following cumulative exposure to Propofol and Thiopental (100 µg/mL and 200 µg/mL) compared with untreated control cells (0 µg/mL). Statistically significant changes (*p* < 0.05) are indicated in bold. (Complete amino acid profiles are provided in Supplementary Table S2).

## Data Availability

The datasets used during the current study are available from the corresponding author on reasonable request.

## Supplementary Materials

The following supporting information can be downloaded at: https://www.mdpi.com/article/10.3390/ph18081221/s1. Table S1. Metabolomic Effects of Propofol and Thiopental on Carnitine Metabolism in AML12 Hepatocytes. Table S2. Metabolomic Effects of Propofol and Thiopental on Amino Acid Metabolism in AML12 Hepatocytes. Table S3A. LC-MS/MS Parameters for the Quantitative Analysis of Carnitine and Acylcarnitine Metabolites in AML12 Hepatocytes. Table S3B. LC-MS/MS Parameters for Quantification of Intracellular Amino Acids in AML12 Hepatocytes.
