# Supplementary material for: Anesthetic-Induced Disruption of Amino Acid and Carnitine Profiles: A Metabolomic Comparison of Propofol and Thiopental in Hepatocytes
Source: Pharmaceuticals (Basel). 2025 Aug 19;18(8):1221. doi: 10.3390/ph18081221 (PMC12389001; doi:10.3390/ph18081221)
Supplement: Supplementary file 1 [file pharmaceuticals-18-01221-s001.zip › pharmaceuticals-3779830-Supplementary-Table- S1.pdf]

Table S1. Metabolomic Effects of Propofol and Thiopental on Carnitine Metabolism in AML12 Hepatocytes

	Control		Propofol					Thiopental					Propofol vs Thiopental	
dose	0 µg		100 µg		200 µg			100 µg		200 µg			100 µg	200 µg
	Mean	Std. Deviation	Mean	Std. Deviation	Mean	Std. Deviation	p-value	Mean	Std. Deviation	Mean	Std. Deviation	p-value	p-value	p-value
C0	0.4115	0.0151	0.1477	0.0032	0.0862	0.0026	**<0.001**	0.1452	0.0073	0.2403	0.0045	**<0.001**	0.625	**<0.001**
C2	0.8540	0.0183	0.5362	0.0072	0.5081	0.0078	**<0.001**	0.3631	0.2064	0.7415	0.0226	**0.006**	0.220	**<0.001**
C3	0.1938	0.0068	0.1404	0.0090	0.1390	0.0076	**<0.001**	0.1225	0.0820	0.1779	0.0088	0.238	0.726	**0.004**
C4	0.0426	0.0051	0.0428	0.0027	0.0319	0.0097	0.142	0.1576	0.0907	0.0284	0.0023	**0.044**	0.093	0.575
C4DC	0.0036	0.0015	0.0032	0.0011	0.0015	0.0009	0.146	0.0071	0.0068	0.0020	0.0017	0.366	0.389	0.694
C5	0.0310	0.0020	0.0264	0.0070	0.0242	0.0020	0.228	0.0432	0.0334	0.0202	0.0069	0.415	0.478	0.392
C5_1	0.0050	0.0007	0.0039	0.0039	0.0038	0.0017	0.814	0.0199	0.0130	0.0032	0.0029	0.071	0.110	0.780
C5OH	0.0637	0.0051	0.0578	0.0054	0.0502	0.0046	**0.046**	0.0581	0.0590	0.0551	0.0051	0.954	0.992	0.286
C5DC	0.0065	0.0057	0.0038	0.0033	0.0021	0.0036	0.488	0.0150	0.0131	0.0057	0.0026	0.387	0.225	0.228
C6	0.0109	0.0053	0.0095	0.0068	0.0071	0.0024	0.684	0.0407	0.0263	0.0049	0.0036	0.064	0.118	0.426
C6DC	0.0039	0.0034	0.0051	0.0019	0.0058	0.0025	0.699	0.0129	0.0167	0.0044	0.0023	0.503	0.505	0.522
C8	0.0060	0.0016	0.0075	0.0023	0.0022	0.0015	**0.032**	0.0376	0.0092	0.0037	0.0036	**0.001**	**0.005**	**0.541**
C8_1	0.0084	0.0043	0.0050	0.0038	0.0057	0.0019	0.494	0.0149	0.0160	0.0054	0.0029	0.510	0.354	0.885
C8DC	0.0063	0.0050	0.0029	0.0022	0.0042	0.0022	0.516	0.0203	0.0163	0.0057	0.0050	0.223	0.141	0.652
C10	0.0127	0.0063	0.0090	0.0050	0.0066	0.0032	0.384	0.0404	0.0175	0.0078	0.0019	0.020	0.040	0.618
C10_1	0.0044	0.0038	0.0088	0.0010	0.0031	0.0034	0.131	0.0438	0.0348	0.0055	0.0037	0.091	0.156	0.457
C10DC	0.0359	0.0005	0.0289	0.0016	0.0239	0.0004	**<0.001**	0.0216	0.0211	0.0234	0.0029	0.361	0.582	0.778
C12	0.0017	0.0030	0.0031	0.0008	0.0002	0.0004	0.215	0.0202	0.0133	0.0004	0.0007	**0.038**	0.156	0.741
C14	0.0077	0.0034	0.0055	0.0028	0.0052	0.0043	0.668	0.0366	0.0379	0.0020	0.0021	0.200	0.292	0.306
C14_1	0.0044	0.0007	0.0040	0.0033	0.0014	0.0009	0.217	0.0327	0.0172	0.0022	0.0020	0.017	0.097	0.583
C14_2	0.0082	0.0037	0.0039	0.0029	0.0037	0.0007	0.152	0.0262	0.0244	0.0041	0.0020	0.212	0.253	0.749
C16	0.0148	0.0019	0.0084	0.0013	0.0110	0.0006	**0.004**	0.0161	0.0122	0.0079	0.0029	0.391	0.384	0.203
C16_1	0.0029	0.0023	0.0040	0.0014	0.0018	0.0013	0.343	0.0195	0.0095	0.0008	0.0009	**0.013**	**0.049**	0.350
C18	0.0114	0.0006	0.0081	0.0009	0.0085	0.0009	0.004	0.0141	0.0198	0.0067	0.0021	0.736	0.648	0.241
C18_1	0.0084	0.0008	0.0049	0.0030	0.0031	0.0020	0.056	0.0149	0.0122	0.0037	0.0011	0.228	0.241	0.668
C18_2	0.0043	0.0014	0.0030	0.0020	0.0017	0.0010	0.178	0.0097	0.0076	0.0032	0.0008	0.239	0.262	0.113
C18_1_OH	0.0015	0.0014	0.0015	0.0007	0.0020	0.0009	0.794	0.0042	0.0060	0.0012	0.0011	0.578	0.525	0.391

Intracellular concentrations (mean ± SD) of carnitine and acylcarnitine species in AML12 hepatocytes following exposure to cumulative doses of Propofol and Thiopental (100 µg and 200 µg), compared to untreated controls (0 µg). Statistically significant changes ($p < 0.05$) are highlighted. Propofol-treated cells were used as the reference group for comparative analyses.
