# Supplementary material for: Anesthetic-Induced Disruption of Amino Acid and Carnitine Profiles: A Metabolomic Comparison of Propofol and Thiopental in Hepatocytes
Source: Pharmaceuticals (Basel). 2025 Aug 19;18(8):1221. doi: 10.3390/ph18081221 (PMC12389001; doi:10.3390/ph18081221)
Supplement: Supplementary file 1 [file pharmaceuticals-18-01221-s001.zip › pharmaceuticals-3779830-Supplementary-Table- S2.pdf]

Table S2. Metabolomic Effects of Propofol and Thiopental on Amino Acid Metabolism in AML12 Hepatocytes

	**Control**		**Propofol**					**Thiopental**					**Propofol vs Thiopental**	
dose	**0 µg**		**100 µg**		**200 µg**			**100 µg**		**200 µg**			**100 µg**	**200 µg**
	Mean	Std. Deviation	Mean	Std. Deviation	Mean	Std. Deviation	p-value	Mean	Std. Deviation	Mean	Std. Deviation	p-value	p-value	p-value
ALANINE	19.75	1.30	7.63	0.50	6.58	0.43	**<0.001**	7.72	0.51	8.20	0.54	**<0.001**	0.851	**0.015**
ARGININE	20.37	1.43	4.93	0.35	13.50	0.94	**<0.001**	0.77	0.05	5.90	0.41	**<0.001**	**0.002**	**<0.001**
ASPARAGINE	7.58	0.57	2.28	0.17	2.01	0.15	**<0.001**	2.49	0.19	3.15	0.23	**<0.001**	0.228	**0.002**
ASPARTIC_ACID	6.77	0.54	2.42	0.19	2.18	0.17	**<0.001**	3.46	0.28	3.47	0.28	**<0.001**	**0.006**	**0.002**
CITRULLINE	3.71	0.31	2.63	0.22	1.21	0.10	**<0.001**	4.04	0.34	3.22	0.27	**0.046**	**0.004**	**0.000**
GLUTAMINE	556.80	50.05	323.50	29.08	687.39	61.78	**<0.001**	428.34	38.50	711.47	63.95	**0.002**	**0.020**	0.663
GLUTAMIC_ACID	42.59	4.06	13.73	1.31	11.92	1.14	**<0.001**	13.99	1.33	13.15	1.25	**<0.001**	0.823	0.277
GLYCINE	16.54	1.67	7.60	0.77	7.55	0.76	**<0.001**	7.55	0.76	9.41	0.95	**<0.001**	0.949	0.057
HISTIDINE	3.46	0.37	1.66	0.18	2.21	0.24	**0.001**	2.20	0.23	2.51	0.27	**0.005**	**0.034**	0.220
LEUCINE	25.07	2.83	10.01	1.13	14.46	1.63	**<0.001**	11.85	1.33	13.66	1.54	**<0.001**	0.142	0.570
ISOLEUCINE	19.58	2.32	8.48	1.01	13.66	1.62	**0.001**	12.62	1.50	15.00	1.78	**0.011**	**0.017**	0.388
ALLOISOLEUCINE	0.42	0.05	0.16	0.02	0.23	0.03	**<0.001**	0.22	0.03	0.25	0.03	**0.001**	**0.031**	0.381
LYSINE	19.72	2.58	7.66	1.00	10.23	1.34	**<0.001**	9.61	1.26	11.02	1.44	**0.001**	0.104	0.529
METHIONINE	2.65	0.86	1.42	0.46	1.75	0.57	0.136	1.71	0.55	1.91	0.62	0.288	0.533	0.760
ORNITHINE	1.11	0.37	0.77	0.26	0.94	0.31	0.476	2.97	0.99	2.39	0.80	0.059	**0.020**	**0.043**
PHENYLALANINE	3.43	1.18	1.44	0.49	1.83	0.63	0.053	1.76	0.60	2.25	0.77	0.137	0.511	0.500
PROLINE	9.68	3.42	3.83	1.35	4.02	1.42	**0.033**	5.29	1.87	6.20	2.19	0.17	0.335	0.222
SERINE	5.66	2.06	2.62	0.95	2.95	1.07	0.08	3.01	1.10	3.80	1.38	0.185	0.665	0.450
THREONINE	9.40	3.52	4.15	1.56	6.14	2.30	0.117	5.42	2.03	6.83	2.56	0.28	0.438	0.746
TRYPTOPHAN	2.12	0.29	1.04	0.14	1.28	0.18	**0.002**	1.21	0.17	1.32	0.18	**0.005**	0.263	0.783
TYROSINE	8.96	1.29	3.90	0.56	5.23	0.75	**0.001**	4.88	0.70	5.91	0.85	**0.005**	0.134	0.352
VALINE	11.18	2.14	4.57	0.87	5.43	1.04	**0.003**	5.42	1.04	5.78	1.11	**0.006**	0.339	0.704
ALPHAAMINOADIPIC_ACID	0.08	0.02	0.04	0.01	0.06	0.01	**0.035**	0.03	0.01	0.08	0.02	**0.005**	0.062	0.193
ALPHAAMINOPIMELIC_ACID	0.57	0.10	0.49	0.09	0.47	0.08	0.373	0.48	0.09	0.46	0.08	0.312	0.986	0.852
ANSERINE	6.10	1.12	5.78	1.07	6.09	1.12	0.926	6.71	1.24	6.63	1.22	0.797	0.379	0.599
ALPHAAMINOBUTYRIC_ACID	0.22	0.09	0.11	0.04	0.09	0.03	0.068	0.14	0.06	0.19	0.08	0.478	0.421	0.102
BETAAMINOISOBUTYRIC_ACID	0.00	0.00	0.13	0.03	0.29	0.07	**0.001**	0.18	0.04	0.26	0.06	**0.001**	0.192	0.700
GAMMAMINOBUTYRIC_ACID	0.53	0.13	0.00	0.00	0.35	0.09	**0.001**	0.01	0.00	0.39	0.10	**0.001**	**0.007**	0.611
BETA_ALANINE	0.49	0.12	0.23	0.06	0.22	0.06	**0.014**	0.22	0.06	0.27	0.07	**0.02**	0.853	0.399
SARCOSINE	1.44	0.37	0.63	0.16	0.49	0.13	**0.007**	0.62	0.16	0.67	0.18	**0.014**	0.902	0.227
CYSTATHIONINE	0.28	0.08	0.09	0.02	0.10	0.03	**0.004**	0.08	0.02	0.14	0.04	**0.007**	0.761	0.223
THIAPROLINE	1.09	0.30	0.40	0.11	0.50	0.14	**0.012**	0.18	0.05	0.25	0.07	**0.001**	**0.035**	0.050
METHYLHISTIDINE_1	0.00	0.00	0.03	0.01	0.03	0.01	**0.004**	0.00	0.00	0.04	0.01	**<0.001**	**0.004**	0.407
METHYLHISTIDINE_3	0.01	0.00	0.02	0.00	0.00	0.00	**0.014**	0.00	0.00	0.00	0.00	**0.001**	**0.005**	**0.016**
HYDROXYLYSINE	0.20	0.06	0.01	0.00	0.10	0.03	**0.003**	0.01	0.00	0.01	0.00	**0.001**	0.658	**0.008**
HYDROXYPROLINE	4.77	1.50	1.93	0.61	2.64	0.83	**0.037**	2.22	0.70	2.58	0.81	0.051	0.619	0.941
CYSTINE	2.61	0.39	0.20	0.03	1.56	0.23	**<0.001**	0.40	0.06	1.03	0.15	**<0.001**	**0.007**	**0.030**
HISTAMINE	0.00	0.00	0.01	0.00	0.00	0.00	**0.001**	0.00	0.00	0.01	0.00	**0.004**	**0.024**	**0.002**
ETANOLAMINE	0.75	0.15	0.19	0.04	0.51	0.10	**0.002**	0.35	0.07	0.22	0.05	**0.002**	**0.029**	**0.012**
PHOSPHOETANOLAMINE	26.37	5.63	7.23	1.54	6.84	1.46	**0.001**	2.38	0.51	5.88	1.26	**<0.001**	**0.007**	0.438
OH_TRP_5	0.08	0.02	0.10	0.02	0.27	0.06	**0.002**	0.01	0.00	0.42	0.09	**<0.001**	**0.002**	0.067
TAURINE	10.01	2.29	23.10	5.28	7.04	1.61	**0.003**	4.41	1.01	4.60	1.05	**0.007**	**0.004**	0.093

Intracellular concentrations (mean ± SD) of amino acids in AML12 hepatocytes following exposure to cumulative doses (100 µg and 200 µg) of Propofol and Thiopental, compared with untreated control cells (0 µg). Statistically significant changes ($p < 0.05$) are indicated.
