# Supplementary material for: Anesthetic-Induced Disruption of Amino Acid and Carnitine Profiles: A Metabolomic Comparison of Propofol and Thiopental in Hepatocytes
Source: Pharmaceuticals (Basel). 2025 Aug 19;18(8):1221. doi: 10.3390/ph18081221 (PMC12389001; doi:10.3390/ph18081221)
Supplement: Supplementary file 1 [file pharmaceuticals-18-01221-s001.zip › pharmaceuticals-3779830-Supplementary-Table- S3B_Amino Acids .pdf]

Supplementary Table S3B. LC-MS/MS Parameters for Quantification of Intracellular Amino Acids in AML12 Hepatocytes

Metabolite	Q1 > Q3 (m/z)	Retention Time (min)
ALANINE	218.10 > 31.20	6.657
ARGININE	303.20 > 70.10	3.571
ASPARAGINE	243.30 > 112.20	4.914
ASPARTIC_ACID	304.20 > 216.20	9.397
CITRULLINE	304.00 > 156.00	4.422
GLUTAMINE	275.10 > 130.20	4.282
GLUTAMIC_ACID	318.20 > 230.20	10.01
GLYCINE	204.30 > 764.20	5.342
HISTIDINE	370.20 > 196.20	9.421
LEUCINE	260.20 > 172.20	11.879
ISOLEUCINE	260.20 > 130.20	11.936
ALLOISOLEUCINE	260.20 > 200.30	11.477
LYSINE	361.20 > 170.10	10.043
METHIONINE	278.20 > 142.20	9.097
ORNITHINE	347.30 > 156.20	8.656
PHENYLALANINE	294.00 > 164.00	11.654
PROLINE	244.10 > 156.20	8.828
SERINE	234.30 > 104.20	4.774
THREONINE	248.10 > 74.10	5.567
TRYPTOPHAN	33.20 > 245.20	10.379
TYROSINE	396.20 > 222.20	14.18
VALINE	246.20 > 158.20	9.725
ALPHAAMINOADIPIC_ACID	332.20 > 98.00	10.8
ALPHAAMINOPIMELIC_ACID	346.20 > 198.20	13.696
ANSERINE	369.20 > 309.20	10.509
ALPHAAMINOBUTYRIC_ACID	232.20 > 144.20	7.668
BETAAMINOISOBUTYRIC_ACID	232.20 > 130.20	10.269
GAMMAMINOBUTYRIC_ACID	232.20 > 86.20	7.535
BETA_ALANINE	218.20 > 98.00	6.487
SARCOSINE	218.20 > 116.20	6.086
CYSTATHIONINE	479.20 > 230.00	13.596
THIAPROLINE	262.10 > 174.20	11.901
METHYLHISTIDINE_1	298.20 > 210.10	5.024
METHYLHISTIDINE_3	298.10 > 96.00	4.895
HYDROXYLYSINE	317.20 > 171.20	4.711
HYDROXYPROLINE	260.20 > 172.20	11.9
CYSTINE	497.20 > 248.20	13.311
HISTAMINE	284.10 > 137.90	9.023
ETANOLAMINE	148.10 > 62.10	3.155
PHOSPHOETANOLAMINE	288.10 > 88.20	3.161
OH_TRP_5	349.10 > 261.20	7.621
TAURINE	212.10 > 126.20	2.61

LC-MS/MS analysis was performed using a reverse phase amino acid analytical column maintained at 30 °C with a flow rate of 0.2500 mL/min. A 0.5 µL aliquot of each sample was

injected, and chromatographic separation was completed in 19 minutes per sample. The mobile phase consisted of 65% Mobile Phase A and 35% Mobile Phase B. Detection was conducted using a tandem mass spectrometer equipped with an electrospray ionization (ESI) source operating in positive ion mode. The ion source parameters were as follows: gas temperature 300 °C, gas flow 10 L/min. and nebulizer pressure 3.0 L/min.
