# Supplementary material for: Anesthetic-Induced Disruption of Amino Acid and Carnitine Profiles: A Metabolomic Comparison of Propofol and Thiopental in Hepatocytes
Source: Pharmaceuticals (Basel). 2025 Aug 19;18(8):1221. doi: 10.3390/ph18081221 (PMC12389001; doi:10.3390/ph18081221)
Supplement: Supplementary file 1 [file pharmaceuticals-18-01221-s001.zip › pharmaceuticals-3779830-Supplementary-Table- S3A_Carnitine.pdf]

Supplementary Table S3A. LC-MS/MS Parameters for the Quantitative Analysis of Carnitine and Acylcarnitine Metabolites in AML12 Hepatocytes

Metabolite	Q1 > Q3 (m/z)	Collision Energy (CE)	Polarity
C0	TIC	-	Positive
C2	TIC	-	Positive
C3	274.20 > 85.00	20	Positive
C4	288.20 > 85.00	22	Positive
C5	302.20 > 85.00	24	Positive
C5:1	300.20 > 85.00	26	Positive
C5OH	318.20 > 85.00	27	Positive
C5DC	388.30 > 85.00	28	Positive
C6	316.20 > 85.00	30	Positive
C8	344.20 > 85.00	31	Positive
C8:1	342.20 > 85.00	32	Positive
C4DC	374.30 > 85.00	33	Positive
C10	372.30 > 85.00	34	Positive
C10:1	370.30 > 85.00	35	Positive
MethylGlutaryl	401.30 > 85.00	36	Positive
C6DC	402.30 > 85.00	37	Positive
C12	400.30 > 85.00	39	Positive
C14	428.40 > 85.00	40	Positive
C14:1	426.40 > 85.00	41	Positive
C14:2	424.40 > 85.00	42	Positive
C8DC	430.40 > 85.00	43	Positive
C16	456.40 > 85.00	44	Positive
C16:1	454.40 > 85.00	46	Positive
C10DC	458.40 > 85.00	47	Positive
C18	484.40 > 85.00	48	Positive
C18:1	482.40 > 85.00	49	Positive
C18:2	480.40 > 85.00	50	Positive
C18:1 OH	498.40 > 85.00	51	Positive

A 3 µL aliquot of each sample was injected into the LC-MS/MS system. Chromatographic separation was performed at a column temperature of 30 °C with a flow rate of 0.1000 mL/min, and each run was completed within 3 minutes. The mass spectrometric detection was conducted using a tandem mass spectrometer equipped with an electrospray ionization (ESI) source operating in positive mode. Ion source parameters were set as follows: gas temperature, 250 °C; drying gas flow, 15 L/min; nebulizer pressure, 3.0 L/min. The mobile phase consisted of Mobile Phase A (100%) and Mobile Phase B (0%), as per system specifications. Metabolite-specific MRM transitions, Q1/Q3 ion pairs, and collision energies are detailed in the table.
